# Genomic amplification of *MYC* as double minutes in a patient with APL-like leukemia

**DOI:** 10.1186/s13039-014-0067-6

**Published:** 2014-10-22

**Authors:** Pino J Poddighe, Hans Wessels, Pauline Merle, Marisa Westers, Shama Bhola, Anne Loonen, Sonja Zweegman, Gert J Ossenkoppele, Marielle J Wondergem

**Affiliations:** Department of Clinical Genetics, VU University Medical Center, De Boelelaan 1117, PK 0X011, Amsterdam, 1081 HV The Netherlands; Department of Haematology, VU University Medical Center, Amsterdam, The Netherlands

**Keywords:** Acute promyelocytic leukemia, *MYC*, Double minutes, Cytogenetics, SNP-array

## Abstract

**Background:**

Acute promyelocytic leukemia (APL) is a subtype of acute myeloid leukemia (AML) characterized by a *PML-RARA* fusion due to a translocation t(15;17). Its sensitivity to treatment with all-trans retinoic acid (ATRA), which causes differentiation of the abnormal promyelocytes, combined with anthracycline based chemotherapy makes it the best curable subtype of acute myeloid leukemia. A rapid and accurate diagnosis is needed in the first place to prevent (more) bleeding problems. Here we present a patient with a leukemia with an APL-like morphology but no detectable *PML-RARA* fusion, as demonstrated by RT-PCR and cytogenetic analysis.

**Results:**

Unexpectedly, karyotyping revealed numerous double minutes (dmins). Fluorescence in situ hybridization (FISH) with DNA probes specific for the *MYC*-region showed the presence of multiple *MYC* amplicons. SNP-array analysis uncovered amplification of the 8q24.13-q24.21 region, including the *MYC*-gene, flanked by deletions in 8q24.13 and 8q24.21-q24.22, and a homozygous deletion in 9p21.3, flanked by heterozygous deletions in the same chromosome region.

**Conclusions:**

The diagnosis was revised to AML, not otherwise specified (AML, NOS) and therefore therapy with ATRA was discontinued.

## Background

Acute promyelocytic leukemia (APL) is a hematological emergency frequently associated with severe coagulation disturbances. The typical morphology shows abnormal, usually bilobed hypergranular promyelocytes. In some cases the cytoplasmic granules are so large or numerous that they completely fill the cell, obscuring the nuclear cytoplasmic limit. Frequently characteristic cells containing bundles of Auer rods, the so-called faggot cells, are seen. In APL, Sudan Black (SB) or myeloperoxidase (MPO) is always strongly positive in all blast cells, with the reaction product covering the whole cytoplasm and often the nucleus too [1].

Cytogenetically a reciprocal translocation t(15;17)(q24;q21) is present, leading to a fusion gene consisting of the proximal part of the promyelocytic leukemia gene (PML) on 15q24 and the distal part of the retinoic acid receptor alpha (*RARA*) gene usually on 17q21 [2]. This has therapeutic impact, since APL with a t(15;17) has a particular sensitivity to treatment with all-trans retinoic acid (ATRA). Treatment with ATRA, combined with cytotoxic chemotherapy or arsenic trioxide (ATO) results in complete remission rates of over 90% [3]. The high morbidity and mortality associated with the coagulation abnormality already present in most patients at diagnosis requires that ATRA must be initiated immediately after the diagnosis is suspected.

Rare cases of APL with typical morphology lack the classic translocation in routine cytogenetic studies. They may still express the *PML-RARA* transcript due to a cryptic *PML-RARA* fusion gene e.g. as a result of an insertion of the *RARA* gene near the *PML* gene on 15q24. These APL have the same response to ATRA as the classical or hypergranular APL.

Another subgroup of APL, the so-called variant or hypogranular APL, has the typical translocation t(15;17) and is also ATRA responsive, but usually presents with a leukocytosis, which may increase quickly. In these hypogranular cases the characteristic cells are not promyelocytes but bilobed blasts with seemingly absent granules and infrequent faggot cells and a strong positive SB or MPO reaction [4].

In the present study we present a case that morphologically resembled a classic APL and was treated as such. The diagnosis had to be reconsidered when additional investigations showed an unexpected cytogenetic result.

## Case presentation

**Figure 1:**
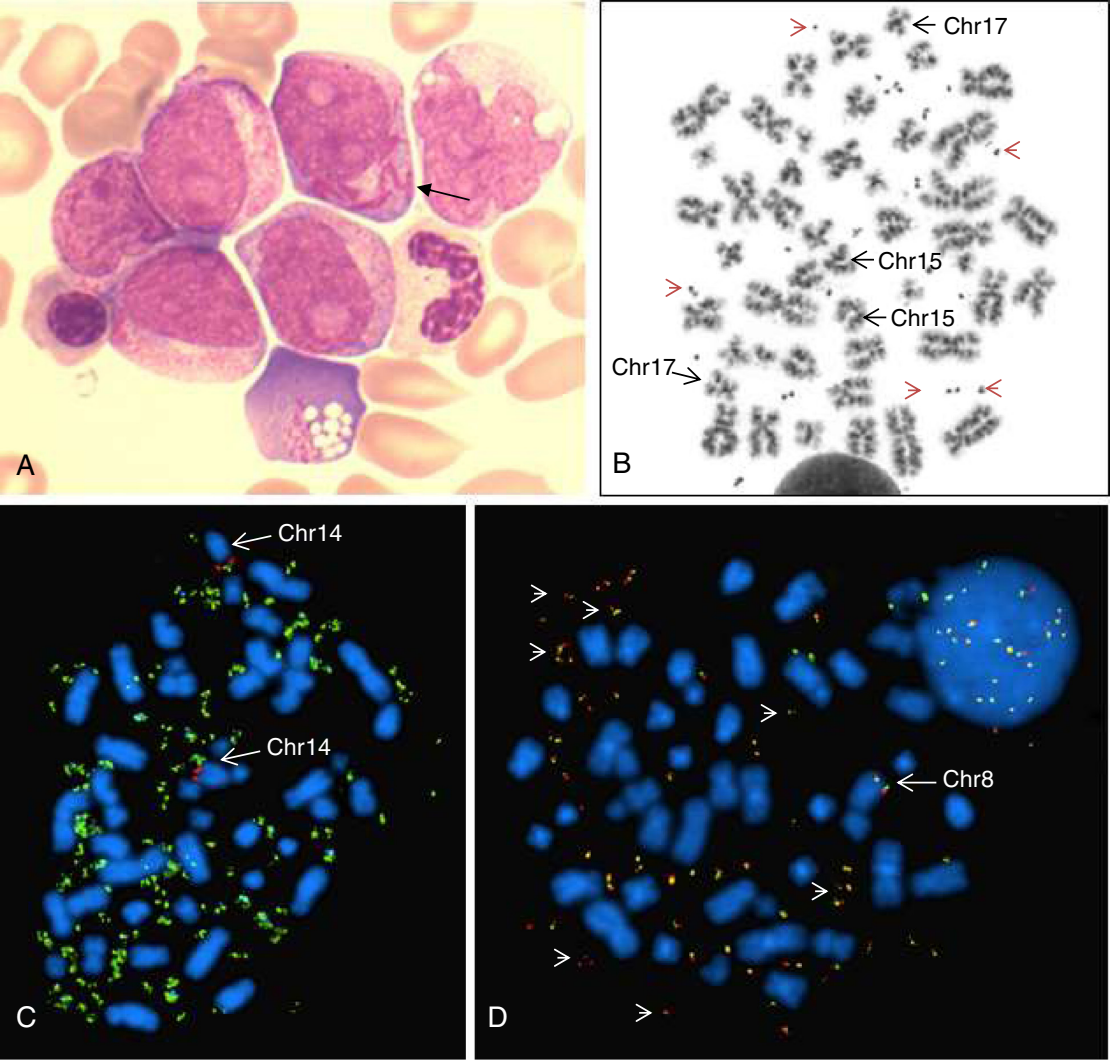
**Cytomorphologic and cytogenetic results. (A)** A representative cytomorphologic bone marrow field, showing blasts with Auer rods (arrow) and azurophilic inclusions. **(B)** A representative metaphase cell demonstrating normal chromosomes 15 and 17, and more than 20 double minutes (red arrowheads). **(C)** Metaphase FISH of this patient with the ON MYC(green)/IGH(red) t(8;14) Fusion Probe (Kreatech), showing *MYC*-positive dmins (green), and both chromosomes 14 (arrow); in this cell it was not possible to indicate the normal chromosome 8 due to the high number of dmins **(D)** Metaphase FISH with a BAC-probe RP1-80K22 (base pair position 128,667,455-128,814,588) for *MYC* gene (red) and a flanking BAC-probe RP11-125A17 (base pair position 128,865,417-129,036,660; green), demonstrating multiple copies of dmins (red-green signals). Only one chromosome 8 (arrow) contains the *MYC* gene region.

A 76-year-old man presented with exertional dyspnea, visual disturbances, night sweats and progressive fatigue. His medical history showed chronic obstructive pulmonary disease, Diabetes Mellitus type 2, hypercholesterolemia and alcohol abuse. On physical examination he had some petechiae and hematoma on the lower extremities. No lymphadenopathy or organomegaly was found. His white blood cell count was extremely elevated (220 × 10^9^/l). Hemoglobin was 6.5 mmol/l and platelets were 20 × 10^9^/l. The peripheral blood smear revealed 37 × 10^9^/l blasts, with Auer rods, but also 78 × 10^9^/l promyelocytes, with some faggot cells, resembling typical APL (Figure 1A). Other abnormal laboratory findings included a LDH of 5230 U/l and creatinine of 130 micromol/l. No coagulation abnormalities were present.

Since he had signs of leukostasis (dyspnea, visual disturbances and tinnitus) he was immediately treated with leukapheresis in addition to the administration of daunorubicin (45 mg/m^2^) and, because of the typical APL morphology, ATRA (45 mg/m^2^ in two doses per day).

## Methods

### Immunophenotyping

Flow cytometric analysis of the peripheral blood was performed according to standard guidelines for immunophenotyping of acute leukemia with some modifications (www.cytometrie.nl). An eight-color antibody panel was applied; data were acquired on a FACSanto II flow cytometer and analyzed with FACS-DIVA software (BD Biosciences, San José, CA, USA).

### Cytogenetic studies

The patient’s peripheral blood was set up in two 24-hour RPMI 1640 cultures, one unstimulated and one stimulated with growth factors G/CSF, IL3 and GM/CSF. After standard cytogenetic harvesting and GTG banding 20 metaphase cells were analyzed from the stimulated culture. The karyotype was described according to ISCN 2013 [5].

Fluorescence in situ hybridization (FISH) using directly labeled probes was performed according to the manufacturer's instructions in combination with our established laboratory protocol. The following probes were used: Vysis LSI PML/RARA Dual Color Dual Fusion Probe (Abbott Molecular IL, Hoofddorp, The Netherlands), ON MYC/IGH t(8;14) Fusion Probe (Kreatech, Amsterdam, The Netherlands), and the BAC probes RP11-150N13 [Chr8:126,376,029-126,557,325 Mb(hg19)], RP11-495D4 [Chr8:126,530,000-126,729,672 Mb(hg19)], RP11-367L7 [Chr8:128,459,593-128,628,028 Mb(hg19)], RP1-80K22 [Chr8:128,667,455-128,814,588 Mb(hg19)], RP11-125A17 [Chr8:128,865,417-129,036,660 Mb(hg19)], RP11-316E19 [Chr8:133,852,795-134,111,178 Mb(hg19)] and RP11-268C19 [Chr8:134,410,759-134,585,494 Mb(hg19)] from BlueGnome Ltd, Cambridge, UK.

### Molecular diagnostics

RNA was isolated from mononuclear blood cells, using RNAeasy (Qiagen). cDNA synthesis of 1 µg RNA was performed using M-MLV (Invitrogen) and random hexamer primers (Roche). *PML-RARa* fusion transcripts were amplified using RT-PCR according to Miller et al. [6]. PCR products were analyzed on agarose gel. FLT3-ITD RT-PCR followed by GeneScan analysis was done as described previously [7].

### Genomic profiling and data analysis

Microarray-based genomic profiling was carried out with 250 ng DNA isolated from peripheral blood using the CytoScan HD array platform (Affymetrix, Inc., Santa Clara, CA, USA) and was performed according to the manufacturer’s protocol. The data obtained by the CytoScan HD array platform were analyzed using Nexus copy number software (Biodiscovery Inc., Hawthorne, CA, USA) and annotations of genome version GRCh37 (hg19).

## Results

Immunophenotyping showed a cell population that was CD34 negative and positive for CD117, HLA-DR, MPO and CD15, with heterogeneous expression of CD33+ and weak expression of CD13, CLIP expression was absent, and a second cell population with more monocytic characteristics: positive for CD11b, CD11c, and aberrantly CD56.

Karyotyping revealed an abnormal male karyotype, but instead of a translocation t(15;17) the cells showed 7 to 50 dmins (see Figure 1B): 46,XY,7~50dmin[20]. Interphase FISH with the LSI PML/RARA DC DF probe showed no *PML-RARA* fusion in 200 cells (results not shown). Molecular analysis revealed that both *PML-RARa* fusion and *FLT3* were negative. Since double minutes in AML have mostly been described as consisting of *MYC*, we performed FISH with the DNA probe ON MYC/IGH t(8;14) Fusion Probe. This probe indeed showed that the dmins stained positive for *MYC* (Figure 1C). Furthermore, the MYC-probe signal was only present on one chromosome 8, indicating loss of the *MYC* region from the other chromosome 8 (see Figure 1D). The karyotype was therefore adjusted: 46,XY,7-50dmin[20].ish del(8)(q24.2q24.2)(MYC-), dmin(MYCx7~50)[10].

**Figure 2:**
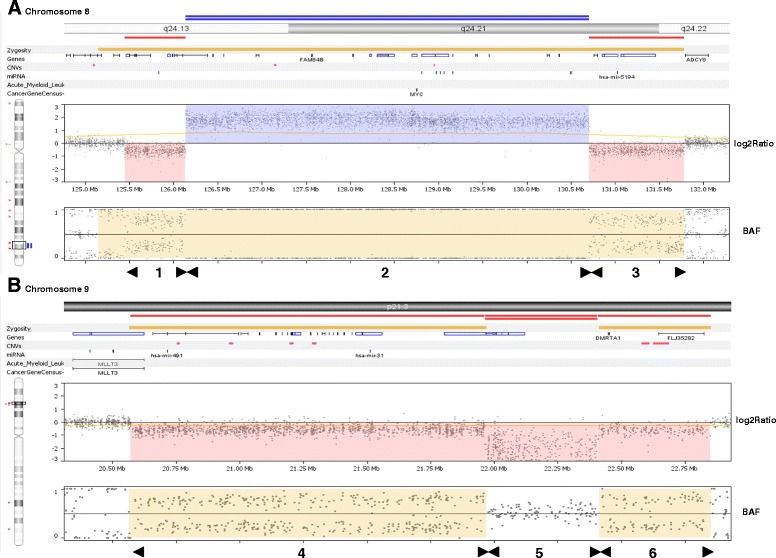
**SNP-array results for chromosome regions 8q24 (A) and 9p21.3 (B). (A)** The log2 ratio demonstrate that amplification of the MYC-region of 4.57 Mb (section 2) is flanked by a proximal deletion of 693 kb (section 1) and a distal deletion of 1,07 Mb (section 3). **(B)** The homozygous deletion of 483 kb (section 5) is flanked by a distal heterozygous deletion of 1.39 Mb (section 4) and a proximal deletion of 423 kb (section 6). BAF = biallelic frequency.

Genome-wide SNP array analysis revealed an amplification of 4,56 Mb on 8q24.13-q24.21, which contained 15 genes, including *MYC*, flanked by a proximal deletion at 8q24.13 of 693 kb (10 genes) and a distal deletion at 8q24,21 of 1,07 Mb (6 genes) (see Figure 2A and Table 1). On 9p21.3 a deletion of 2,28 Mb (31 genes) was observed, of which 442 kb was homozygous (see Figure 2B and Table 1).

**Table 1:** **Overview of genomic aberrations detected by SNP-array**

**Chromosome region**	**Genomic position**	**Length (base pairs)**	**Copy number**	**Gene symbol**
8q24.13q24.13	125,444,383-126,137,622	693.240	Deletion	TRMT12, RNF139, TATDN1, NDUFB9, MTSS1, LOC157381, ZNF572, SQLE, KIAA0196, NSMCE2
8q24.13q24.21	126,137,622-130,706,799	4.569.178	Amplification	NSMCE2, TRIB1, LOC100130231, FAM84B, PCAT1, POU5F1B, LOC727677, MYC, MIR1204, MIR1205, PVT1, MIR1206, MIR1207, MIR1208, LOC728724
8q24.21q24.22	130,706,799-131,782,618	1.075.820	Deletion	GSDMC, FAM49B, MIR5194, LOC100507117, ASAP1, ASAP1-IT1
9p21.3p21.3	20,571,017-21,966,002	1.394.986	Deletion	MLLT3, MIR491, FOCAD, PTPLAD2, IFNB1, IFNW1, IFNA21, IFNA4, IFNA7, IFNA10, IFNA16, IFNA17, IFNA14, IFNA22P, IFNA5, KLHL9, IFNA6, IFNA13, IFNA2, IFNA8, IFNA1, IFNE, MIR31HG, MIR31, MTAP
9p21.3p21.3	21,966,002-22,408,814	442.813	Homozygous deletion	C9orf53, CDKN2A, CDKN2B, CDKN2B-AS1
9p21.3p21.3	22,408,814-22,851,531	442.718	Deletion	DMRTA1, FLJ35282

Additional FISH with the probe combination RP1-80K22 (overlapping the *MYC*-gene) and a distal flanking BAC-probe RP11-125A17 confirmed that the dmins contained more than the *MYC*-gene (see Figure 1D). The proximal flanking DNA probes RP11-150N13, RP11-495D4 and RP11-367L7 were also present on the dmins, whereas the distal flanking DNA probes RP11-316E19 and RP11-268C19 were absent (results not shown).

The final karyotype we decided on was 46,XY,7-50dmin[20].ishdel(8)(q24.2q24.2)(MYC-),dmin(MYCx7~50)[10].arr 8q24.13(125,444,383-126,137,622)x1,8q24.13q24.21 (126,137,622-130,706,799)amp,8q24.21q24.22(130,706,799-131,782,618)x1,9p21.3(20,571,017-21,966,002)x1,9p21.3(21,966,002-22,408,814)x0,9p21.3(22,408,814-22,851,531)x1.

Following leukapheresis and administration of daunorubicin leucocyte counts dropped to 0.9 10^9^/l. Based on the additional investigations the diagnosis of APL was altered to AML, not otherwise specified (AML, NOS) [4].

The patient’s age and physical condition didn’t allow to deliver intensive anti-leukemic chemotherapy. He subsequently developed a paralytic ileus and hypotension and died.

## Conclusions

In this report we describe a patient with a rare APL-like phenotype. At presentation the peripheral blood smears showed a typical APL morphology. However, immunophenotyping results were not typical for APL, that is usually HLADR negative with homogeneous expression of CD33 and CLIP positive [8]. Moreover, the extreme leukocytosis and also the absence of abnormal coagulation tests did not fit in with the diagnosis of APL. In hypogranular APL the leucocyte count can be very high with a rapid doubling time, but the patient’s cell morphology and immunophenotyping didn’t resemble a hypogranular APL. The additional investigations confirmed this; there was no detectable *PML-RARA* fusion product present. In fact, cytogenetic analyses showed no t(15;17), but the presence of 7 to 50 double minutes (dmins). SNP-array identified a 4,56 Mb large amplicon (containing 15 genes), flanked by a proximal deletion of 693 kb (10 genes) and a distal deletion of approximately 1,07 Mb (6 genes), as well as a homozygous deletion in 9p21.3 of 443 kb (4 genes), which was flanked by two heterozygous deletions: one distal deletion of about 1,4 Mb (25 genes) and one proximal deletion of 443 kb (2 genes), see Table 1.

Amplification of human chromosome region 8q24 has been associated with many types of solid tumors, such as breast, prostate, colon, lung, ovaries and pancreas [9-11]. The genes in this region are mostly all oncogenes or tumor suppressor genes, affecting general cancer susceptibility. In general, the *MYC*-gene is involved in this amplification and can be present as dmins or homogeneously staining regions (hsr). Gene amplification in AML is rare, the most frequent gene involved being *MYC* and the second most common oncogene MLL [12], mostly as part of a complex karyotype. *MYC* amplification manifesting as dmins has been described in only a few cases with an APL or APL-like morphology [13-15].

In solid tumors, such as colon, pancreatic and breast carcinomas, brain tumors and neuroblastomas, dmins can be observed as a late genomic event in tumorigenesis, the dmins being associated with a rapid cellular growth and poor prognosis. In AML dmins appear less frequently, in about 1% of the cases [12,16,17]. The mechanism of the excision of DNA segments from an otherwise intact chromosome, followed by circularization and amplification by mutual recombination to produce dmins, has been described by Carroll et al. [18]. Storlazzi et al. [19] have provided evidence for this so-called episomal model for the formation of MYC-containing dmin in acute myeloid leukemia.

The deletion in 8q was larger than the amplified segment in dmins, and this phenomenon was also observed in a study by Storlazzi et al. [20], who describe a chromosomal deletion in 8q24 corresponding to or larger than the amplicon in 68% of 34 investigated AML/MDS cases, suggesting post-replicative excision of DNA followed by circularization (episome) as the mechanism behind the dmin formation.

The first patient with MYC amplification in dmins as the sole cytogenetic aberration has been described by Frater et al. [15]. Also in our patient the excision of *MYC* from 8q24 with subsequent amplification of this region into dmins may have led to upregulation of the expression of the *MYC* oncogene, a known critical nuclear transcription factor.

Deletions of 9p are not frequent recurrent chromosome aberrations in AML. Usvasalo et al. [21] reported a study in which multiple areas of copy number loss, or homozygous loss within a larger heterozygous loss region, in 9p was restricted to ALL patients, and was not observed in AML patients. Interestingly, the APL-like patient without a *PML-RARA* fusion presented by Bruyère et al. [13] showed *MYC* amplification as dmins and an apparently terminal deletion of the short arm of chromosome 9 with the breakpoint at band p21. Our findings support their suggestion that there might be an association between loss of 9p, dmins, and an APL-like morphology.

In summary, we describe a patient with an APL-like morphology, who showed no *PML-RARA* fusion but *MYC* amplification in dmins. It is of utmost importance to have a rapid confirmation or exclusion of t(15;17) in an acute leukemia that morphologically resembles APL. Therefore, in our routine practice we have implemented a rapid, four hours interphase FISH test using a LSI PML/RARA DC DF Probe (Vysis) on bone marrow or blood smears from patients suspected for APL. In contrast to the other similar reported case [15] our patient died soon after diagnosis from abdominal sepsis before proper treatment could be initiated.

## Consent

Informed consent was obtained from the patient for the publication of this report and any accompanying images.

## Abbreviations

AML NOS: Acute myeloid leukemia, not otherwise specified
APL: Acute promyelocytic leukemia
ATO: Arsenic trioxide
ATRA: All-trans retinoic acid
dmins: Double minutes
BAC: Bacterial artificial chromosome
FISH: Fluorescence in situ hybridization
MPO: Myeloperoxidase
PML: Promyelocytic leukemia gene
RARA: Retinoic acid receptor alpha gene
SB: Sudan Black
SNP: Single nucleotide polymorphism
